# *Neuronal Signaling*: A reflection on the Biochemical Society’s newest journal and an exciting outlook on its next steps

**DOI:** 10.1042/NS20210007

**Published:** 2021-02-08

**Authors:** Aideen M. Sullivan, S. Clare Stanford

**Affiliations:** 1 Department of Anatomy and Neuroscience, and The APC Microbiome Institute, University College Cork, Cork, Ireland; 2 Department of Neuroscience, Physiology and Pharmacology, University College London, London, U.K.

**Keywords:** neurodegeneration, neuroscience, signalling

## Abstract

The inaugural Editor-in-Chief of *Neuronal Signaling*, Aideen M. Sullivan, reflects on the journal’s journey so far and welcomes the new Editor-in-Chief, Clare Stanford, as she shares some of the exciting initiatives and plans for its future.

*Neuronal Signaling* was launched in 2017 by the Biochemical Society, through its publisher Portland Press, to address the need for a dedicated journal for research and reviews on the molecular, cellular and systematic signaling processes involved in the development, functioning and disorders of the nervous system. Over the past couple of decades, there have been significant advances in our understanding of the mechanisms by which cells communicate within the nervous system. The mission of *Neuronal Signaling* is to provide an interdisciplinary home for neuroscientific research, covering all aspects of *in vitro* and *in vivo* signaling, from cell to brain in health and disease. Figure 1 shows a snapshot selection of *Neuronal Signaling* journal cover images, representing some of the excellent and innovative work that has been published in the journal.

**Figure 1:**
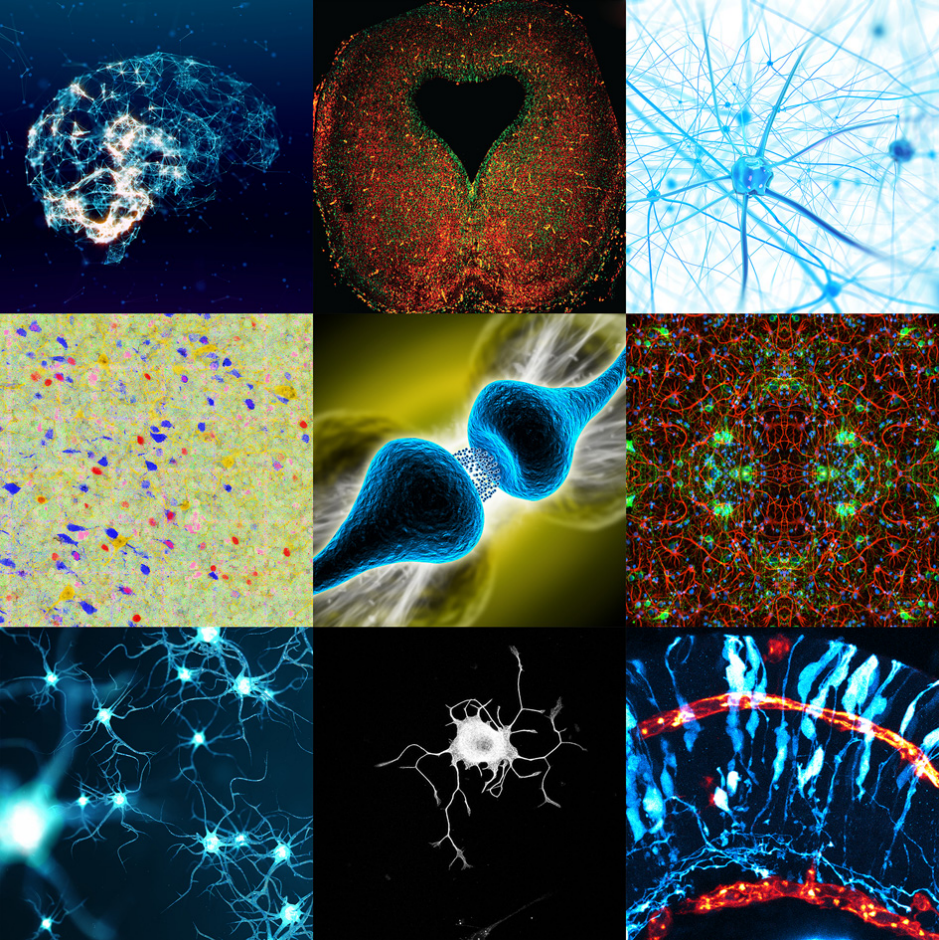
A snapshot selection of *Neuronal Signaling* journal cover images

It has been an honor to act as Editor-in-Chief of *Neuronal Signaling* during its first 4 years. I have had the pleasure of working with an excellent Editorial Board, the members of which hold international reputations and expertise in many aspects of nervous system development and physiology, as well as neurodegenerative, neurological and psychiatric disorders. Over the past 4 years, the Editorial Board has continued to expand, achieving growth and diversity in terms of both expertise, geographic location and gender.

The partnership between the Biochemical Society and Portland Press is key to the success of *Neuronal Signaling*. Founded in 1911, the Biochemical Society is the U.K.’s largest single-discipline learned society and its mission is to advance the molecular biosciences. Portland Press is committed to open scholarship and to open access publishing. It works in collaboration with the Publications Committee of the Biochemical Society to ensure the delivery of high-quality, peer-reviewed content in their suite of journals. Portland Press supports the scholarly and charitable activities of the Biochemical Society and enhances its global reach and service to the international research community. As a Portland Press journal, *Neuronal Signaling* has benefitted from the vast experience and success of its sister journals in the portfolio, including the *Biochemical Journal* and *Clinical Science*.

In March 2019, *Neuronal Signaling* launched a collaboration with ScienceOpen, a platform committed to open science via facilitating open and public communication between academics. This collaborative platform has further strengthened the impact of the journal on the neuroscientific research community and will be enhanced by the ongoing work of the expert Editorial Board.

In May 2020, *Neuronal Signaling* achieved the significant milestone of being indexed in PubMed and PubMed Central, a crucial step for the journal’s growth and discoverability. PubMed Central indexing, alongside Google Scholar and the Directory of Open Access Journals (DOAJ), provides a measure of the quality and importance of the articles that are published in *Neuronal Signaling*, and enhances their availability to, and discoverability by, the scientific community and the general public.

To reflect on her outlook for the journal’s future, I am delighted to hand over to Professor Clare Stanford, the incoming Editor-in-Chief of *Neuronal Signaling* as of January 2021.

*Neuronal Signaling* has achieved a great deal, over the last 4 years, and we are keeping up the momentum in planning the further development of the journal. Some initiatives are already underway, while others are on the horizon, but they will all need conscientious support from the scientific community.

Although Impact Factors attract considerable scepticism and even overt criticism, it is inevitable that they are regarded as a benchmark for a journal’s perceived status in the scientific community. Our successful application for listing in PubMed Central means that achieving indexing on Web of Science (and achieving an Impact Factor) is now our next objective, through sustaining our efforts to encourage submission of high-quality papers.

The Biochemical Society and Portland Press have set up ‘*Read & Publish*’ agreements with several influential institutions, which will certainly help us to achieve our objectives. These ‘*Read & Publish*’ agreements enable authors, from participating institutions, to publish open access in *Neuronal Signaling* without needing to pay any author-facing article publishing charges (APCs). This is part of the Biochemical Society’s open scholarship strategy and will provide a real incentive for authors to put *Neuronal Signaling* on their list of first-choice journals when deciding where to send their manuscripts.

*Neuronal Signaling* is also increasing its journal profile though collaborations with other groups. The Biochemical Society is a partner society for the forthcoming British Neuroscience Association Festival of Neuroscience, and *Neuronal Signaling* will be sponsoring a symposium on ‘*From stem cells to whole animals: the scope and appraisal of research models in vitro and in vivo’.* It is a pity that this will have to be a virtual meeting, but the quality of the science will not be affected. The Biochemical Society will be hosting a virtual booth for *Neuronal Signaling* at that meeting, so please do visit to find out more about the scope of the journal, seek advice for submission of articles and learn about our plans for its development. We hope to arrange more collaborations of that kind, in the future.

In addition to these projects, *Neuronal Signaling* is planning a series of themed collections of original and review articles in 2021. We will be inviting submissions for a topical collection on olfaction, including themes such as the mechanisms behind the loss of sense of smell (hyposmia) in relation to COVID-19, Parkinson’s disease and Kallmann syndrome, as well as nasal drug delivery and sexual dimorphisms in olfactory signaling. We are also excited to launch a collection based on the sponsored BNA symposium, mentioned above, which will be entitled ‘*Emerging technologies for research models of neuronal human disorders in vivo and in vitro’.* Dr Tom Cunningham (Harwell, U.K.), one of my fellow speakers at the symposium, has kindly agreed to act as Co-Guest Editor for this *Neuronal Signaling* collection. We encourage readers to offer their own suggestions, for more themed topics to be featured in *Neuronal Signaling*, especially if they would like to act as a Guest Editor for the collection.

We are aware that all these activities will generate more work for everyone involved with running the journal. To ensure that the processing of manuscripts remains timely and efficient, we shall be looking to expand the Editorial Board. We already have distinguished editors from the U.K., Ireland, Australia, mainland Europe and U.S.A., all of whom have made invaluable contributions to the journal’s success. *Neuronal Signaling* will be looking to recruit additional Editorial Board members, especially from the other continents, to ensure we maintain a truly international profile. We are proud that 60% of the Editorial Board are women and we shall strive to maintain a gender-balanced team through judicious recruitment of new members.

The Biochemical Society is proud to support early-career researchers (ECRs) through many initiatives including their new Biochemistry Focus webinar series dedicated to ECRs. I was delighted to take part in chairing the successful first webinar of 2021 on ‘Developments in Neuroscience’, where we heard from four gifted ECRs who shared some of their innovative current work in neuroscience with the biosciences community. Full details on past webinars and the exciting programme for 2021 are available on the Biochemical Society webinars page.

As well as all these initiatives, we need to keep abreast of developments in the scientific community, more generally. As a first step, we shall be revising our guidelines to authors, to offer some advice on the reporting of experiments that use animal models of complex, human neuronal disorders, with the aim of trying to improve their successful translation into humans. We shall also keep abreast of developments in research practice, particularly in respect of integrity and open science. As part of that process, we shall be reviewing the ARRIVE 2.0 guidelines [1] and highlighting factors that are particularly pertinent for readers and authors of this journal. Look out for more editorials about those topics in the near future.

As Professor Aideen Sullivan demits as Editor-in-Chief, I must thank her, the Editorial Board and everyone at Portland Press for all their efforts and resounding success with this fledgling journal. Fortunately, Aideen will be carrying on as an Associate Editor and I look forward to working with this outstanding team of professionals and building on their progress over the next 4 years.

Finally, we hope this editorial will encourage you to read some of the excellent work being published in *Neuronal Signaling* [2–21]. Of course, we would welcome any pre-submission queries and look forward to receiving your manuscripts in the future.

## Open Access

Open access for this article was enabled by the participation of University College London in an all-inclusive *Read & Publish* pilot with Portland Press and the Biochemical Society under a transformative agreement with JISC.

## Abbreviation

ECR: early-career researcher
